# Spinal epidural abscess caused by *Pasteurella multocida* mimicking aortic dissection: a case report

**DOI:** 10.1186/s12879-019-4097-x

**Published:** 2019-05-22

**Authors:** Koji Oh, Takafumi Inoue, Toshihiko Saito, Chihiro Nishio, Hiroki Konishi

**Affiliations:** 1 Department of General Internal Medicine, Kobe City Medical Center West Hospital, Hyogo, Japan; 2 Department of Orthopedics, Kobe City Medical Center West Hospital, Hyogo, Japan

**Keywords:** *Pasteurella multocida*, Zoonosis, Bacteremia, Spinal epidural abscess

## Abstract

**Background:**

*Pasteurella multocida* (*P. multocida*) forms part of the normal flora of many animals. Although it is a common causative agent of skin and soft tissue infection after an animal bite or scratch, in rare cases it can cause spinal infections in humans.

**Case presentation:**

A 68-year-old immunocompetent woman presented with fever and sudden onset of severe back pain mimicking aortic dissection. No findings related to the pain were revealed on enhanced computed tomography or initial magnetic resonance imaging (MRI) of the spine. The patient was found to be bacteremic with *P. multocida*, although she had no apparent injury related to animal contact. Repeated evaluation by MRI with gadolinium-contrast established the diagnosis of spinal epidural abscess. The patient was cured by the rapid initiation of antimicrobial therapy without surgery.

**Conclusions:**

We describe the successful treatment of an individual with a spinal epidural abscess due to *P. multocida* without surgery. *P. multocida* infections may occur as sudden presentations. Obtaining the patient history of recent animal contact is essential. Repeated MRI evaluation may be required when spinal infections are suspected. To the best of our knowledge, this is the first report which describes a case of spinal epidural abscess due to this organism.

## Background

*Pasteurella multocida* (*P. multocida*) is present in the oral, nasopharyngeal, and upper respiratory tract microbiota among cats, dogs, and other domestic or wild animals [1]. The organism is a common causative agent of skin and soft tissue infections (SSTI) following animal bites or scratches, and in rare cases, it can cause spinal infections [2, 3]. Here, we present an interesting case of spinal infection mimicking aortic dissection. This is the first case report of a spinal epidural abscess due to *P. multocida*.

## Case presentation

A 68-year-old woman visited our emergency department (ED) because of a sudden attack of severe back pain. The pain developed so suddenly that the ED physician provided a tentative diagnosis of aortic dissection. Chest and abdominal computed tomography (CT) with contrast enhancement revealed no findings related to the pain and both aortic dissection and any aortic involvement were excluded. The ED physician prescribed non-steroidal anti-inflammatory drugs. The next day, the patient presented to the outpatient department and was admitted for further examination. She had medical histories of asthma and atrial fibrillation, was not receiving any medication, was a current smoker, and was not an alcohol consumer. She owned a corgi dog that lived in her house and she had been bitten and scratched by the dog daily.

Upon admission, her body temperature was 37.5 °C, with blood pressure 127/48 mmHg, heart rate 84 beats per minute and oxygen saturation 95% in ambient air with no accelerated respiration. She looked very ill suffering from the severe back pain. A physical examination did not note spinal knocking pain, neurological abnormality, or any other specific findings. A laboratory test revealed a white blood cell count of 13,360 cells/mm^3^ (normal value: 3900–9800 cells/mm^3^), platelet count of 17.0 × 10^4^/μL, serum creatinine level of 0.66 mg/dL, and C-reactive protein level of 18.5 mg/dL (normal value: 0–0.5 mg/dL). Re-performed enhanced CT and plane magnetic resonance imaging (MRI) of the spine was not diagnostic.

On the second hospital day, Gram-negative bacilli were detected in her blood cultures. We started intravenous meropenem 1 g every 8 h, based on a provisional diagnosis of spinal epidural abscess or vertebral osteomyelitis/discitis. *P. multocida* was identified, and drug susceptibility was confirmed by the Vitek2 system with GN and AST-N228 card, bioMérieux (Table 1) [4]. Then, the antimicrobial treatment was switched to intravenous ampicillin 2 g every 6 h.

**Table 1:** Identification and drug susceptibility results

*Pasteurella multocida*		
	MIC (μg/ml)	Susceptibility
Ampicillin	<=2	S
Piperacillin	<=4	S
Sulbactam/Ampicillin	<=2	S
Tazobactam/Piperacillin	<=4	S
Cefazoin	<=4	S
Ceftazidime	<=1	S
Ceftriaxone	<=1	S
Cefepime	<=1	S
Cefmetazole	<=1	S
Aztreonam	<=1	S
Imipenem	<=0.25	S
Meropenem	<=0.25	S
Amikacin	4	S
Tobramycin	<=1	S
Ciprofloxacin	<=0.25	S
Levofloxacin	<=0.12	S
Sulfamethoxazole/Trimethoprim	<=20	S

The test was performed using the Vitek2 system (GN and AST-N228 card, bioMérieux). Gram-negative bacilli were identified as *Pasteurella multocida*. The organism was susceptible to all antimicrobials tested. S: susceptible

**Fig. 1:**
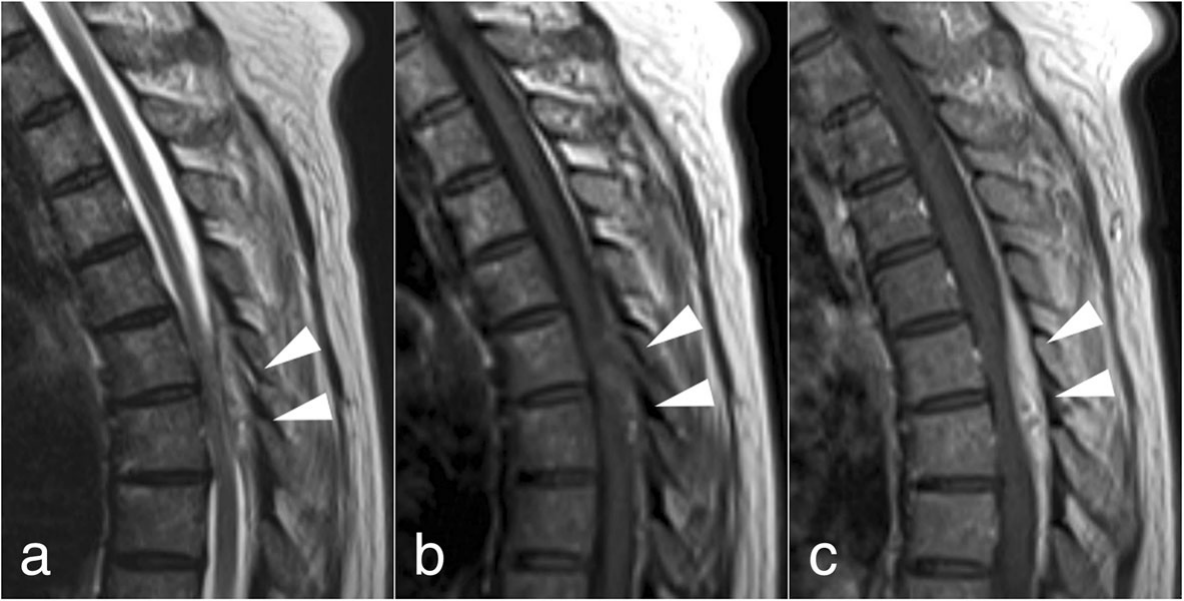
Sagittal MRIs of the thoracic spine on the eighth hospital day. **a** T2-weighted image. **b** T1-weighted image. **c** T1-weighted image with gadolinium enhancement. The posterior epidural abscess extends from the Th5 level to the Th6 level (arrow). Internally, there is a high T2/fluid signal, low T1 and gadolinium-enhancement. An associated mass effect caused spinal canal stenosis and slight compression of the spinal cord, without cord signal changes

On the eighth hospital day, MRI was re-performed with gadolinium-contrast to confirm the diagnosis. T2-weighted imaging and T1-weighted imaging with gadolinium enhancement revealed an epidural abscess at the Th5–6 level (Fig. 1). We decided to withhold a surgery and continued the conservative treatment because of absence of a neurological deficit. On the twenty-fourth hospital day, the abscess had disappeared on the plane MRI. The antimicrobial treatment was switched to oral levofloxacin 500 mg every 24 h before hospital discharge. In total, 12-week antibiotic treatment was completed. She visits our hospital with another medical condition which is unrelated to this episode, and no recurrence occurred in 5 years after the treatment was completed.

## Discussion and conclusions

We present a case of *P. multocida* spinal epidural abscess in an immunocompetent patient who had a dog as a pet. She had an attack of severe back pain mimicking aortic dissection. Repeated evaluation of MRI established the diagnosis of spinal epidural abscess. The patient was cured with the rapid initiation of antimicrobial therapy without surgery.

*P. multocida* is a non-spore forming, non-motile, Gram-negative coccobacillus found in the nasopharynx or gastrointestinal tract of cats, dogs, and other animals [5]. It is most commonly recognized as a cause of SSTI after injury by animal bite [6]. *P. multocida* also causes various diseases such as septic arthritis, osteomyelitis, pneumonia, endocarditis, meningitis, and septicemia [1]. Previous studies reported cases of intracranial epidural abscess (an extensive intracranial region), spinal osteomyelitis and paravertebral abscess, although a spinal epidural abscess due to this organism has not been reported [2, 3, 7].

*P. multocida* can cause central nervous system infection through several mechanisms: direct inoculation via an animal bite, contamination from contiguous infected wounds after trauma or neurosurgery, extension from an adjacent infected site by spread through lymphatics or veins, or bacteremic seeding [8]. The same mechanisms are implicated for a spinal epidural abscess. In this case, the patient was bitten frequently when walking her dog. However, she did not present with even superficial wounds when she developed the disease. This suggests that infections related to this organism can occur just with a play-biting even without injuries from animal contact. The organism has been also reported to be transmitted through animal saliva without biting [6]. In addition, some cases were reported where there was no animal contact [5]. In a retrospective cohort study, *P. multocida* infections without an animal bite were often associated with bacteremia, severe comorbidity(ies), immune-incompetent states, the need for intensive care unit management, and substantial mortality [9].

The diagnosis of a spinal epidural abscess is challenging and there is often a substantial diagnostic delay in ED settings [10]. Clinical findings in patients with spinal epidural abscess may develop within hours to days, or the course may be more chronic, over weeks to months. Spinal pain has not been described as sudden onset similar to cases of aortic dissection, which caused a diagnostic error at the initial visit in this case [11]. *Pasteurella* species are associated with a shorter latency period, which is the time from the bite to the appearance of symptoms, compared with staphylococcal or streptococcal infections. Previous studies reported that 43% of patients who contracted *P. multocida* via infection of wounds experienced the rapid onset of local erythema, warmth, swelling, and tenderness. [12, 13]. The characteristic rapid clinical course of *P. multocida* infections might explain the sudden onset of back pain in this case.

When patients present an acute back pain without findings in imaging studies, we should list spinal infections in differential diagnoses and consider obtaining blood cultures, particularly with fever, chillness, leukocytosis, or elevation of inflammatory markers. But fever is present in only 50% of patients with vertebral osteomyelitis, and 60–70% with spinal epidural abscess [11, 14]. One study reported that fever was presented in only 32% and leukocytosis was presented in only 60% of patients with spinal epidural abscess [15].

Gadolinium-enhanced MRI is the preferred method to detect epidural abscess or vertebral osteomyelitis/discitis because of its high sensitivity and specificity [16]. However, the MRI findings in the early phase of the clinical course can be insignificant or subtle. Therefore, a repeat examination should be considered when the initial MRI finding is not diagnostic for spinal infections [17]. In this case, we did not use a contrast agent for the initial evaluation of MRI. Using a contrast agent may have led to an earlier diagnosis. It is clinically important that a repeated MRI and a gadolinium-enhanced MRI may yield the diagnosis of spinal infections.

As a limitation, we referred to criteria for “Other Non-Enterobacteriaceae” from the Clinical and Laboratory Standards Institute (CLSI) M100, Performance Standards for Antimicrobial Susceptibility Testing, 23rd edition to test for antimicrobial susceptibility. At the period of the case, CLSI M45, Methods for Antimicrobial Dilution and Disk Susceptibility Testing of Infrequently Isolated or Fastidious Bacteria, which includes criteria for *P. multocida*, was not available.

In conclusion, we presented a case that developed *P. multocida* infection without evidence of traumatic animal contact. This diagnosis was challenging for two reasons: the initial MRI showed no significant finding, and the symptoms developed atypically with sudden onset. Repeated MRI examination established the diagnosis of spinal epidural abscess. The rapid presentation may be affected by the organisms’ characteristics. We should obtain the patient history of any animal contact at any time. Spinal infection should be considered even when an initial MRI finding is not obvious.

## Abbreviations

CLSI: Clinical and Laboratory Standards Institute
CT: Computed tomography
ED: Emergency department
MRI: Magnetic resonance imaging
*P. multocida*: *Pasteurella multocida*
SSTI: Skin and soft tissue infections
